# Effect of tDCS targeting the M1 or left DLPFC on physical performance, psychophysiological responses, and cognitive function in repeated all-out cycling: a randomized controlled trial

**DOI:** 10.1186/s12984-023-01221-9

**Published:** 2023-07-26

**Authors:** Hafez Teymoori, Ehsan Amiri, Worya Tahmasebi, Rastegar Hoseini, Sidney Grospretre, Daniel Gomes da Silva Machado

**Affiliations:** 1 Exercise Metabolism and Performance Lab (EMPL), Department of Exercise Physiology, Faculty of Sport Sciences, Razi University, Kermanshah, Iran; 2 EA4660-C3S Laboratory – Culture, Sports, Health and Society, University Bourgogne France- Comte, Besancon, France; 3 Research Group in Neuroscience of the Human Movement (NeuroMove), Department of Physical Education, Federal University of Rio Grande do Norte, Natal, RN Brazil

**Keywords:** Non-invasive brain stimulation, Electromyography, Anaerobic performance, Perceived exertion, Affective States, Circumplex Model of Affect

## Abstract

**Background:**

Despite reporting the positive effects of transcranial direct current stimulation (tDCS) on endurance performance, very few studies have investigated its efficacy in anaerobic short all-out activities. Moreover, there is still no consensus on which brain areas could provide the most favorable effects on different performance modalities. Accordingly, this study aimed to investigate the effects of anodal tDCS (a-tDCS) targeting the primary motor cortex (M1) or left dorsolateral prefrontal cortex (DLPFC) on physical performance, psychophysiological responses, and cognitive function in repeated all-out cycling.

**Methods:**

In this randomized, crossover, and double-blind study, 15 healthy physically active men underwent a-tDCS targeting M1 or the left DLPFC or sham tDCS in separate days before performing three bouts of all-out 30s cycling anaerobic test. a-tDCS was applied using 2 mA for 20 min. Peak power, mean power, fatigue index, and EMG of the quadriceps muscles were measured during each bout. Heart rate, perceived exertion, affective valence, and arousal were recorded two minutes after each bout. Color-word Stroop test and choice reaction time were measured at baseline and after the whole anaerobic test.

**Results:**

Neither tDCS montage significantly changed peak power, mean power, fatigue index, heart rate, affective valence, arousal, and choice reaction time *(p> 0.05)*. a-tDCS over DLPFC significantly lowered RPE of the first bout (compared to sham; *p*_*=*_*0.048, Δ*_*=*_*-12.5%)* and third bout compared to the M1 (*p*_*=*_*0.047, Δ*_*=*_*-12.38%)* and sham (*p*_*=*_*0.003, Δ*_*=*_*-10.5%)*, increased EMG of the Vastus Lateralis muscle during the second *(p*_*=*_*0.016, Δ*_*= +*_*40.3%)* and third bout *(p*_*=*_*0.016, Δ*_*= +*_*42.1%)* compared to sham, and improved the score of color-word Stroop test after the repeated all-out task *(p*_*=*_*0.04, Δ*_*= +*_*147%)*. The qualitative affective response (valence and arousal) was also higher under the M1 and DLPFC compared to the sham.

**Conclusion:**

We concluded that tDCS targeting M1 or DLPFC does not improve repeated anaerobic performance. However, the positive effect of DLPFC montage on RPE, EMG, qualitative affective responses, and cognitive function is promising and paves the path for future research using different tDCS montages to see any possible effects on anaerobic performance.

**Trial registration:**

This study was approved by the Ethics Committee of Razi University (IR.RAZI.REC.1400.023) and registered in the Iranian Registry of Clinical Trials (IRCT id: IRCT20210617051606N5; Registration Date: 04/02/2022).

## Introduction

Neuromuscular fatigue is a complex and multi-dimensional phenomenon that has attracted attention for more than a century [1]. Classically, it has been studied under central and peripheral components. However, it is now accepted that there is a loop in which the central nervous system (CNS) and periphery interact dynamically during physical activity and this interaction determines muscle performance and fatigue [2]. Recent findings have shown that the brain plays a pivotal role in this scenario by receiving and processing varied information coming from the periphery and within the CNS itself, and then, preparing appropriate responses to the working muscles [3, 4]. This includes receiving afferent feedback from the most active organs during exercises such as skeletal muscles, heart, and lungs (particularly, group III and IV mechano- and metabo-sensitive muscle afferents), processing of psychophysiological responses [e.g., perceived exertion (RPE), pleasure-displeasure, arousal, motivation, emotional status], and moderating the changes in the corticospinal excitability of neural circuits [5, 6]. In this perspective, the brain plays a vital role in regulating performance and the amount of effort an exerciser puts in during a physical task.

It has been shown that the prefrontal cortex (PFC) at the top of the motor hierarchy and the primary motor cortex (M1) as a downstream area are two important regions that have been corroborated to contribute the most to physical performance [7]. Indeed, the role of the PFC, in particular the left dorsolateral PFC (DLPFC), in exercise tolerance and termination by regulating motivation, cognitive control, and decision-making has been suggested in previous studies [3, 8, 9]. The M1 also plays a crucial role in physical performance because it directs the neural drive originating in higher brain areas and sends the final command to the motor units [10]. This, along with peripheral factors [11], determines muscles’ recruitment capacity to accomplish a specific physical task [5, 7, 12, 13]. Accordingly, it has been postulated that modulating the activity of these two brain regions could bring about alterations in exercise performance, cognitive function, physiological and psychophysiological responses.

Transcranial direct current stimulation (tDCS) is a neuromodulatory technique that may induce changes in ongoing brain activity and/or change neuronal excitability in a polarity-dependent manner [7, 14, 15]. tDCS has shown promising effects on neuromuscular and whole-body exercise endurance, strength, power output, and cognitive function in healthy populations [5, 16–21]. Interestingly, most of the previous studies have assessed the effect of tDCS either on endurance performance (whole-body exercise or single-joint resistance exercise) or muscle strength/power [7]. However, very few studies have explored the effects of tDCS on different aspects of anaerobic performance, specifically anaerobic activities with repeated nature [22–24].

It is noteworthy that many sporting activities involve short bouts of maximal or all-out effort ranging from a few seconds (5 to 10 s) to less than one minute interspersed with periods of sub-maximal activities in between, also known as anaerobic activity [25–27]. Of particular importance, it has been shown that during repeated anaerobic or all-out exercise, peripheral fatigue develops at early stages and as more bouts are repeated, central mechanisms of fatigue are more involved in the exercise-induced impairment in neuromuscular performance [28]. Interestingly, it has been shown that performing even one bout of the 30-s all-out Wingate test could considerably impair the neural drive (34% reduction) to the working muscle indicating its effect on central components regulating exercise performance [28], which could be accentuated by its repeated performance. These central mechanisms might include reduced corticospinal excitability, and reduced motor drive which might be located in the M1 itself or upstream (i.e., DLPFC) due to the processing of afferent feedback from the periphery, affective states (pleasure-displeasure and arousal), motivation, RPE, emotional responses, pain, mood disturbance, and cognitive control [1, 5–7, 9]. In this context, it has been shown that while tDCS targeting M1 increased mean power in sprint exercise and cognitive performance [22], tDCS targeting the DLPFC did not improve repeated sprint ability in 10 bouts of 30 m running exercise [29]. The effect of tDCS may depend on the stimulation parameters such as the nominal target, electrode position, current intensity, density, duration, and timing of application. However, to the best of our knowledge, very few studies have compared the effect of tDCS montage on exercise performance [30], and no study was performed on repeated anaerobic maximal exercise performance. Moreover, most of the previous studies have investigated the effect of tDCS on sprint-interval performance (repeated sprints with ≤ 10 s in duration) [22–24] in which the phosphagen system is the main energy source [26] while there is also no study investigating the efficacy of tDCS in sporting activities that require speed endurance or speed strength in which repeated sprints with longer duration (15 to 90 s) must be performed. In such activities, the glycolytic system is the predominant source of energy and the resultant metabolic perturbations lead to a high accumulation of metabolic by-products which subsequently increase inhibitory signaling to the CNS [25, 27, 31]. Finally, there is still a dearth of comprehensive studies measuring the effect of tDCS on a wide range of variables related to repeated anaerobic exercise such as neuromuscular (i.e., electromyography; EMG) and psychophysiological parameters (e.g., RPE, affective valence, arousal), and cognitive performance which accentuate the need for further studies in this particular area [22, 29, 32].

Taken together, these raise the question of whether applying tDCS over the brain regions involved in regulating such mechanisms could improve repeated anaerobic performance and whether the tDCS target (i.e., M1 vs. DLPFC) could induce different results. Hence, we aimed to investigate the effects of anodal tDCS over M1 and DLPFC on anaerobic performance in repeated all-out anaerobic exercise, physiological, psychophysiological, and cognitive responses. We hypothesized that both tDCS montages targeting M1 and DLPFC (a) would improve repeated anaerobic performance; (b) increase the electromyographic (EMG) amplitude of the tight muscles; (c) decrease RPE; (d) increase the affective responses; and (e) improve the cognitive performance in repeated all-out cycling task [18, 30, 32–35].

## Methods

### Participants

Fifteen young healthy active males voluntarily participated in this randomized, counter-balanced, double-blind, and sham-controlled study. Participants’ characteristics are presented in Table 1. The sample size was calculated a *priori* using G*Power (Version 3.1.9.2, Kiel, Germany) software as follows: test family = F tests; Statistical test = ANOVA: Repeated measures, within factors; α error probability = 0.05; power (1-β err prob) = 0.80; Effect size f = 0.35 [7], number of groups = 1, number of measurements = 3, Correlation among repeated measures = 0.5, and Non-sphericity ɛ correction = 1. Accordingly, 15 participants were deemed appropriate as the sample size for the present study. The inclusion criteria were (1) healthy men aged between 18 and 30 years, (2) to perform the anaerobic exercise as part of their training routine, and (3) classified as category 3 (HEPA active) according to International Physical Activity Questionnaire–Short Form (IPAQ-SF). The exclusion criteria were: (1) suffering from any cardiovascular, pulmonary, and metabolic diseases, (2) history of seizure, epilepsy, or other neurological diseases, (3) implantable devices or pacemakers in the body, and (4) tobacco, drug, and alcohol consumption. The study was approved by the Institutional Ethics Committee (approval number: IR.RAZI.REC.1400.023) and it was conducted following the declaration of Helsinki. All participants gave their written informed consent to the experimental design of the study. This study was registered in the Iranian Registry of Clinical Trials (IRCT id: IRCT20210617051606N5; Registration Date: 04.02.2022). The first participant was included on 14.02.2022, and the trial was terminated on 17.03.2022 in Kermanshah, Iran.

**Table 1:** General characteristics of the participants

Variables	Mean ± SD (n = 15)
Age _(years)_	22.26 ± 2.5
Body Mass _(kg)_	73.85 ± 9.6
Height _(cm)_	179.4 ± 5.4
Body Mass Index _(kg/m_^2^_)_	22.91 ± 2.4
Body Fat _(%)_	17.5 ± 4.8
Fat Mass _(kg)_	13.3 ± 4.8
Fat-Free Mass _(kg)_	60.5 ± 6.0

### General experimental design

**Fig. 1:**
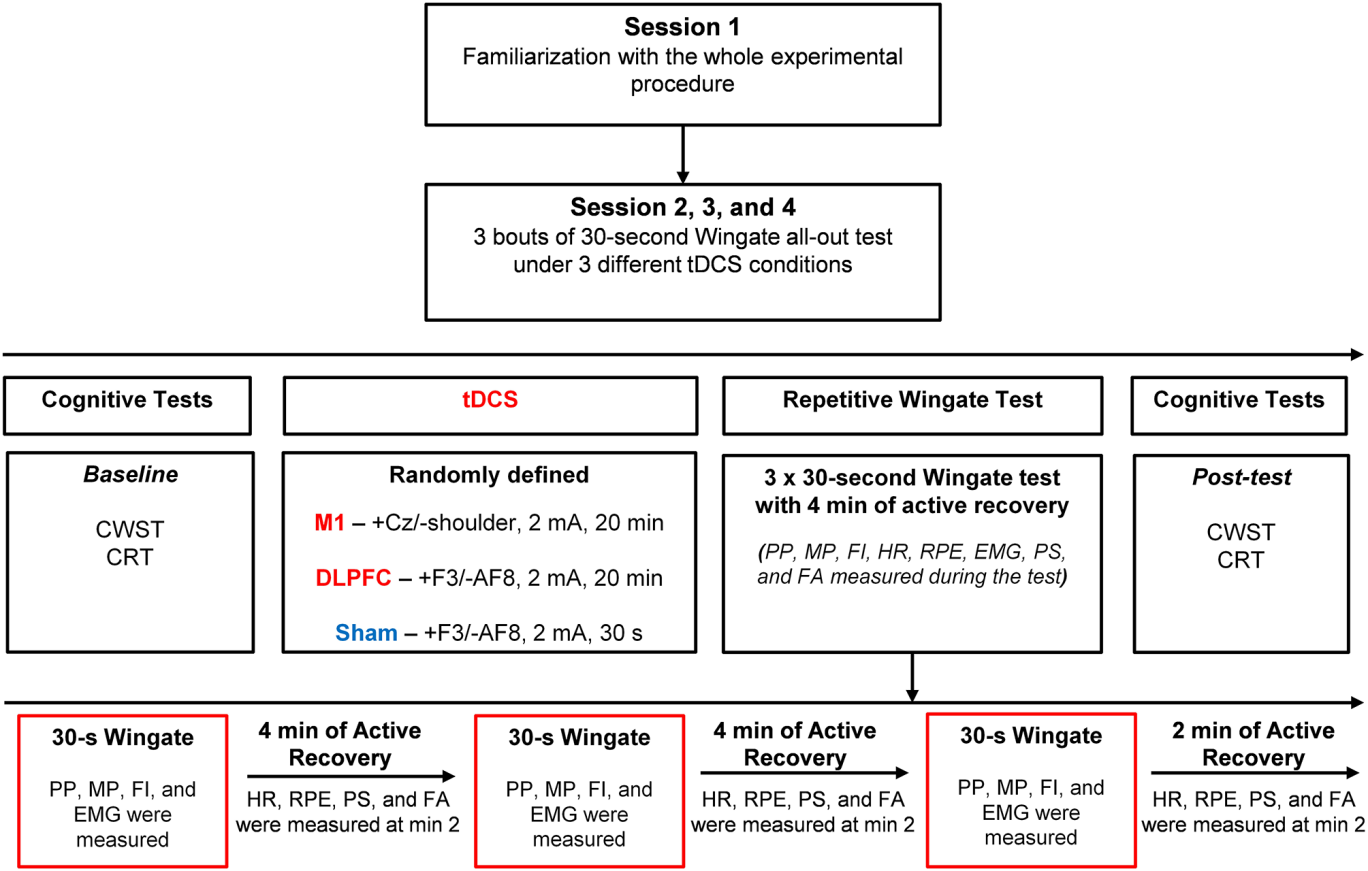
Schematic of the whole study procedure and details of three experimental sessions

Participants came to the laboratory on four different occasions at one-week intervals. The first session was designed for familiarizing the participants with the whole experimental procedure, cycling on the ergometer, brain stimulation, and measuring the study variables. The participants also gave their written informed consent in the first session. From the 2^nd^ to 4^th^ visits, participants first performed the cognitive test and then the maximal isometric voluntary contraction (MIVC) test of knee extensor muscles. Subsequently, participants received tDCS for 20 min in a randomized order (M1, DLPFC, or sham). After tDCS, participants performed 3 bouts of 30-s all-out cycling exercise (i.e., Wingate) interspersed with 4 min of active recovery. During each bout of the Wingate test, physical performance [peak power (PP) and mean power (MP)] and physiological responses (EMG) were measured. During the recovery period after performing each bout, participants reported their psychophysiological responses [RPE and affective states (affective valence and arousal)] and HR was recorded. Finally, two minutes after the third Wingate bout, participants performed again the cognitive tests. Participants and the outcome assessor were blinded regarding the type and site of stimulation in each session (i.e., double-blind design). A 24-hour paper-based dietary recall was applied by a nutrition expert (through an interview with each participant) in the second session (i.e., the first experimental session) and participants were instructed to follow the same diet 24 h before the next two experimental sessions. Moreover, to avoid any effects of circadian rhythm on the study variables, each subject came to the laboratory at the identical time of the day in a laboratory-controlled ambient condition (19–22 °C; 50–60% relative humidity) in all experimental sessions. The whole experimental procedure has been depicted in Fig. 1.

### Randomization, allocation, and concealment

The order of the tDCS conditions was randomized. The randomization was performed by a researcher out of the main research team using the Latin Square method on a publicly available website (www.randomization.com). Blinding was performed by having different researchers applying tDCS (the only one who knew the tDCS condition) and assessing the outcome measures. To do that, when the tDCS intervention was about to start the outcome assessor would leave the room and not come back until the respective tDCS condition was finished, the tDCS device was turned off and the electrodes had been removed from participants’ heads. No information exchange was performed between them. Moreover, participants were not told of which experimental condition they were receiving and the tDCS device was kept behind them (out of sight) and covered so that participants could not see the device and any information on its display.

### MIVC

At the beginning of each experimental session, after cognitive testing, participants performed 3–5 s knee extension MIVC three times with a 150-s rest in between on a custom-made chair with knee and hip fixed at 90° as recommended for Rectus Femoris (RF), Vastus Lateralis (VL), and Vastus Medialis (VM) muscles MIVC test [36]. Verbal encouragement was provided to each participant during the test. During the MIVC test, EMG signals of the VL, VM, and RF muscles were recorded using Surface wireless EMG sensors (Ultium ^TM^ wireless EMG system, Noraxon, Inc., Scottsdale, AZ, USA). EMG signals were amplified (×1.000), high- and low-pass filtered (10 and 500 Hz, respectively), and sampled up to 4000 Hz with the common mode rejection ratio of <-100dB. EMG signals were then registered and analyzed using MyoRESEARCH 3 software (Noraxon, Inc., Scottsdale, AZ, USA) to specify the MIVC of each test. The results corresponding to the best MIVC were used for normalizing the EMG signals of that session.

### Transcranial direct current stimulation (tDCS)

**Fig. 2:**
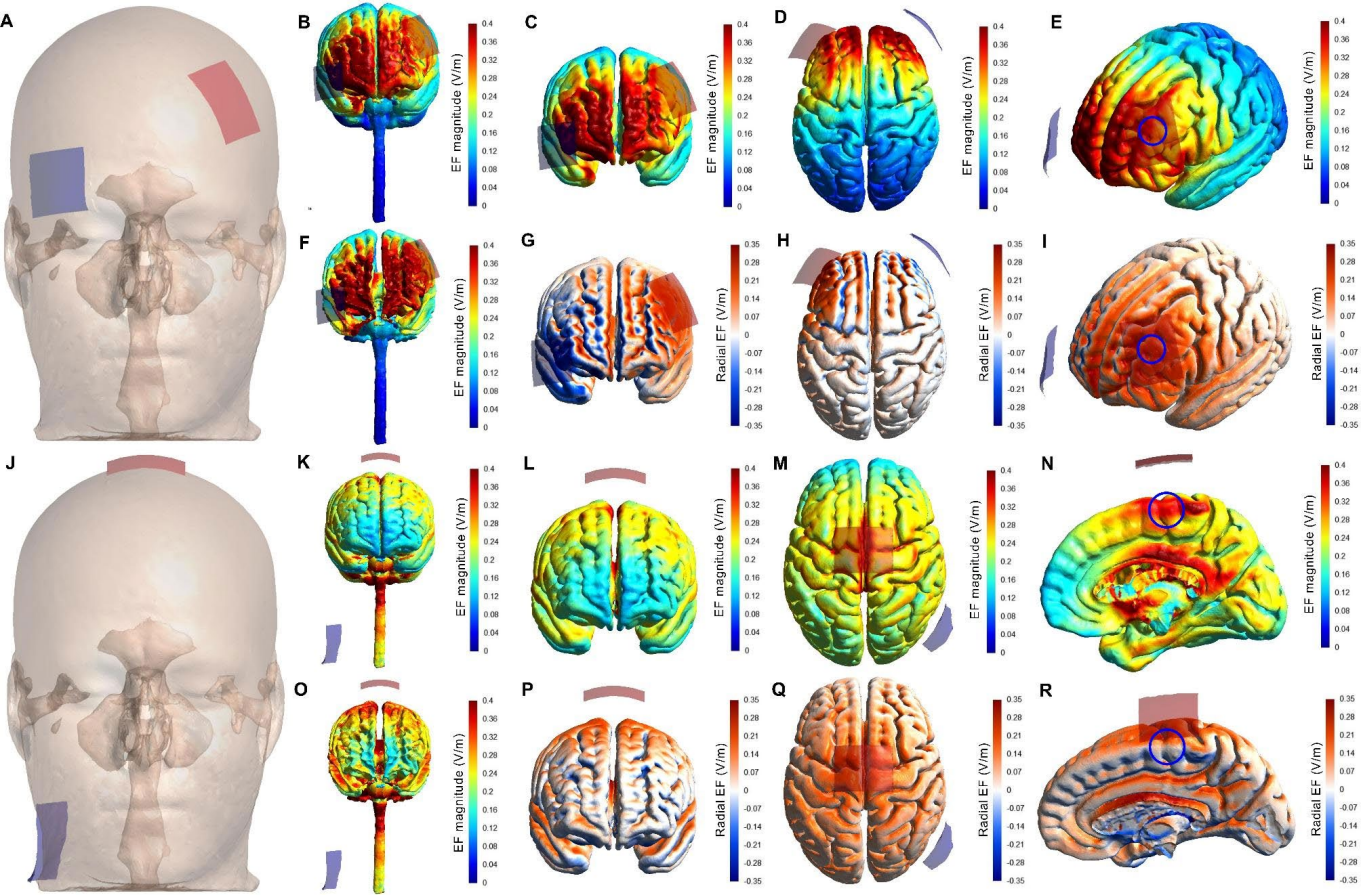
The magnitude and radial component of the electric field induced by tDCS montages. Analysis of tDCS-induced strength and radial (normal to the cortical surface) component of the electric field (EF) using a head model (MNI152) developed from magnetic resonance imaging. Electrode montages targeting anodal tDCS in the left dorsolateral prefrontal cortex (panels **A-I**) and primary motor cortex (panels **J-R**). Anodal (red rectangle; 5 × 4 cm) and cathodal (blue rectangle; 5 × 4 cm) electrodes were placed over the scalp (**A** and **J**). The magnitude of the EF is shown in panels **B-F** and **K-O**, with hot colors (e.g., red) representing stronger EF and cold colors (e.g., blue) representing weaker EF. Panels **G-I** and **P-R** depict the radial EF, with red representing the electric current flowing into the cortex (i.e., inducing excitatory effects) and blue representing the electric current flowing out of the cortex (i.e., inducing inhibitory effects). Panels **E** and **N** show that the research montages reached the target areas with enough electric current magnitude to generate a neuromodulatory effect (blue circles roughly indicating the target locations). Furthermore, as shown in panels **I** and **R**, the target areas were stimulated with the desired polarity (i.e., anodal current) to elicit excitatory effects in the target regions (blue circles roughly indicating the target areas). Because the anatomical model for the M1 tDCS montage does not include the shoulders, the cathode electrode was placed on the lower part of the neck, which provides a good approximation of the shoulder placement. Panels show the EF magnitude and radial components in the gray matter surface (**C-E**, **G-I**, **L-N**, and **P-R**), white matter surface (**F** and **O**), and both (**B** and **K**)

In sessions 2, 3, and 4, and after measuring the cognitive function and MIVC, participants received one of the three tDCS conditions (M1, DLPFC, and sham tDCS) in a randomized order. Participants and the outcome assessor were blinded regarding the type of stimulation in each session (double-blind design). A battery-driven stimulator (NeuroStim 2, Medina Tebgostar, Tehran, Iran) was used to apply tDCS with 2 mA for 20 min. Two carbon electrodes (4 × 5 cm; 20 cm^2^; current density = 0.1 mA/cm²) covered by saline-soaked (NaCl 140 mmol dissolved in Milli-Q water) surface sponges were used as anode and cathode. A 64-channel EEG cap following the international 10–20 EEG system was used to locate target areas over the scalp. For the DLPFC tDCS, the anode was placed over F3 targeting the left DLPFC area, and the cathode was placed over AF8 *(*Fig. 2A*)*. For the M1 tDCS montage, the anode was symmetrically placed over the Cz (2.5 cm on each side of the M1) targeting the motor area of the lower limb, and the cathode was placed over the left shoulder *(*Fig. 2J*)*. The tDCS montages and protocol used in the present study were similar to the study by Etemadi et al. [30]. In M1 and DLPFC conditions, the electric current was gradually ramped up for 30 s, maintained at 2 mA for 20 min, and then progressively ramped down for 30 s. For the sham condition, the DLPFC montage was used but the 2-mA current was maintained active only for 30 s and then was ramped down for 30 s. This sham tDCS procedure has been shown to effectively blind participants [30, 37–39]. Moreover, to avoid creating expectations effects [40, 41], participants were not informed that there would be a sham stimulation condition [30].

### tDCS-induced electric field simulation

The brain current flow during tDCS was calculated using a finite element model following the standard pipeline in SimNIBS 4.0.0 [42]. The magnetic resonance imaging (MRI) MNI 152 head model available in the software was used. MRI data were segmented into surfaces corresponding to the white matter (WM), gray matter (GM), cerebrospinal fluid (CSF), skull, and skin. The electrical conductivities of each segment were determined according to values previously established as follows: WM = 0.126 S/meter (S/m), GM = 0.275 S/m, CSF = 1.654 S/m, bone = 0.010 S/m, and skin/scalp = 0.465 S/m [43], rubber electrode = 29.4 S/m, and saline-soaked sponges = 1.000 S/m. All information concerning the respective tDCS montages was entered into the software: current intensity = 2 mA; electrode position (+ F3/-AF8 and + Cz/-left shoulder); electrode and sponge sizes (5 × 4 cm); electrode thickness = 1 mm; sponge thickness = 5 mm. Because the anatomical model does not include a shoulder for the M1 tDCS montage simulation, the cathode electrode was placed on the lower part of the neck, which provides a good approximation of the shoulder placement. The results of the simulations are presented in Fig. 2, in terms of the electric field magnitude and radial electric field (normal to the cortical surface), both of which are the most important variables for tDCS to exert its neuromodulatory effects [44]. As can be seen in Fig. 2, the montage targeting the left DLPFC reached our nominal target with enough electric field magnitude *(*Fig. 2B, C, D, E, *and F)* with inward current *(*Fig. 2G, H, *and I)*, which is expected to induce neuromodulatory effect [45]. The DLPFC tDCS montage also reached other prefrontal areas between the electrodes such as the bilateral ventromedial and ventrolateral PFC, and right DLPFC *(*Fig. 2*)*. Similarly, the M1 tDCS montage reached the nominal target (motor representation of the lower limbs) with enough strength *(*Fig. 2K, L, M, N, *and O)* and current flow direction *(*Fig. 2P, Q, *and R)* to produce neuromodulatory effect [45]. The M1 tDCS montage also produced large electrical fields in deeper regions of the brain and the spinal cord *(*Fig. 2*)*.

### tDCS-Induced sensations and blinding assessment

To assess participants’ blinding effectiveness, participants completed a questionnaire that lists the sensations and level of intensity experienced during the stimulation after each experimental session [46]. This questionnaire has been used in previous studies involving tDCS and exercise performance [30, 47]. Itching, pain, burning, warmth/heat, pitching, metallic/iron taste, fatigue, and other sensations (open questions) were all listed on the questionnaire. The degrees were none (zero), mild (one), moderate (two), considerable (three), and strong (four). Participants also indicated whether these sensations affected their ability to perform the exercise (0 = not at all; 1 = slightly; 2 = considerably; 3 = much; 4 = very much); when the discomfort started (1 = beginning; 2 = at about the middle; 3 = towards the end); and when it stopped (1 = stopped quickly; 2 = stopped in the middle; 3 = stopped at the end). The “discomfort” generated by tDCS was computed as the sum of the strength scores recorded for all sensations, which ranged from 0 (lack of discomfort) to 28 (maximum discomfort). Considering that the end-of-study corrects guess rates (i.e., % of participants that successfully guessed their experimental condition) might lead to a misleading interpretation of blinding effectiveness [48, 49], it has been suggested to report the “active stimulation guess rate” (i.e., % of participants who guessed they received the active treatment) [48]. Hence, although we reported both correct and active stimulation guesses rates, we considered the latter as the measure of blind effectiveness [48].

### Repeated anaerobic performance (Wingate Test)

A repeated anaerobic performance test, adapted from the Wingate test, was performed after 20 min of tDCS from the 2^nd^ to 4^th^ experimental sessions. Participants performed three bouts of the 30-s cycling all-out Wingate test with 4 min of active recovery on a cycle ergometer (Ergomedic 894E, Monark Sports and Medical, Stockholm, Sweden). The bike settings (saddle height) were kept the same in all experimental sessions, according to each preference set in the familiarization session. A standard 5-minute warm-up was performed, consisting of pedaling with a 2% resistance of total body weight and performing 3 sprints of 5 s at minutes 2, 3, and 4 with a 6.6% resistance of total body weight. After the warm-up and before starting the test, a 3-min passive recovery was applied, and then, with the command “Go”, the participants started pedaling as fast as possible for 30 s against a constant load equal to 7.5% of each participant’s body mass. After each bout of the Wingate test, the participants performed 4 min of active recovery on the bike with 30–40 W at 50 rpm. During the last 10 s of the recovery, the participants were informed to be ready for the next bout and the last 3 s of the recovery phase were counted down by the experimenter to inform the participants of the start point of the next bout of the Wingate test. Strong verbal encouragement was provided during each bout of the Wingate test. The Peak power (PP: highest power output achieved during the 30 s test) and mean power (MP: average power calculated for the complete test duration) were obtained in each bout. The fatigue index was subsequently calculated [FI = (Peak Power - Minimum Power) / (Peak Power × 100)]. It has previously been shown that performing the 30-s all-out Wingate test considerably reduces the neural drive to the working muscles (~ 34%) indicating the high contribution of the CNS in this task [28]. Hence, we assumed that performing 3 bouts of the 30-s all-out Wingate test with 4 min of recovery in between meets the requirements for testing the study’s hypotheses.

### Psychophysiological responses

#### HR

Heart rate was continuously monitored during the whole Wingate test procedure using a chest strap (M430, Polar, Finland) connected to the cycle ergometer. The HR was recorded at min 2 of the 4-min active recovery performed after each bout of the 30-s Wingate test for further evaluation.

#### RPE and Affective States (affective valence and arousal)

RPE was measured using the Borg CentiMax scale (CR100) which ranges from 0 (“nothing at all”) to 100 (“maximal”) [50]. The affective valence (pleasure-displeasure) was measured using the Feeling Scale (FS) comprising 11 items on a spectrum ranging from − 5 (very bad) to + 5 (very good), with zero being neutral [51]. Arousal was measured using the Felt Arousal Scale (FAS) consisting of 6 items scored on a continuum from 1 (low arousal) to 6 (high arousal) [51]. During the familiarization session (first session), participants were acquainted with the concept of perceived exertion, affective valence, and arousal as well as how to report their perceptual responses at specified times. We used the instructions provided elsewhere for measuring the affective states in the present study [52]. Psychophysiological responses were reported at minute 2 of the 4-min active recovery after each bout of the 30-s Wingate test [50].

#### Circumplex model of affect

We constructed the Circumplex Model of Affect (CMA) [53] that consists of a two-dimensional structure that includes the affective valence (pleasure/displeasure) and activation/arousal (low arousal/high arousal) [54–56]. The CMA is presented in four quadrants containing meaningful affective experience: (I) high-activation pleasant affect (upper right) corresponding to an excitement-like state; (II) high-activation unpleasant affect (upper left) corresponding to tension and distress; (III) low-activation unpleasant affect (bottom left), characteristic of boredom and depression; and (IV) low-activation pleasant affect (bottom right), a combination characteristic of calmness and relaxation [54–56].

#### EMG

The EMG of the VL, VM, and RF muscles were recorded during each bout of the 30-s Wingate test. The surface EMG signals were collected strictly according to the recommended standards [57, 58]. Surface wireless EMG sensors (Ultium ^TM^ wireless EMG system, Noraxon, Inc., Scottsdale, AZ, USA) were placed and fixed on the muscle belly of the target muscles of the dominant leg after skin preparation (shaving, abrading, and cleaning with alcohol). EMG signals were amplified (×1.000), high- and low-pass filtered (10 and 500 Hz, respectively), and sampled up to 4000 Hz with the common mode rejection ratio of <-100dB. EMG signals were then registered and analyzed using MyoRESEARCH 3 software (Noraxon, Inc., Scottsdale, AZ, USA) according to EMG amplitude analysis instructions. EMG signals were normalized to the EMG data obtained during the best of three MIVC of the knee extensors for each respective muscle, as described above. The mean value of the EMG amplitude of the VL, VM, and RF muscles (normalized to MIVC) during the Wingate test was recorded and used for statistical analyses.

### Cognitive function measurement

#### Color-word stroop test (CWST)

Inhibitory control involves the ability to control attention, behavior, thoughts, and/or emotions to override an internal predisposition to act automatically (i.e., impulsive), and do what is needed to attain a specific goal [59]. The Stroop test is a standard and valid test for measuring inhibitory control which is considered an important factor in regulating strenuous physical tasks by inhibiting unpleasant sensations during exercise [18]. It has previously been used in other studies with a similar design [18, 22, 30, 60]. The standardized version of the paper-based color-word Stroop test revised by Golden (1975), consisting of 3 cards listing 100 items each presented in a “5 (columns) × 20 (rows)” matrix was used in the present study. The card I (W) included randomly distributed 100 words (red, green, and blue) printed in black ink on a white sheet while no word was followed by itself in a column. Card II (C) consists of 100 colors (written as XXXX) printed in either red, green, or blue ink on a white sheet in which no color was followed by itself in a column or matched the corresponding word position on card (I) Finally, card III (CW) contained 100 colored words on a white sheet in which the order of words from the card I was printed in the order of the colors from card (II). This way no word for a color matched that particular color. The participants were given all three cards with card ‘W’ on top, followed by card ‘C’, then card ‘CW’ placed in front of them on a flat surface. They were instructed to read out loud as many items in each card in 45 s as quickly as they could. If there was a mistake, the experimenter said “No” and the participants had to correct the mistake and continue the test. Moreover, if the participants finished all the columns of each card before 45 s, they were instructed to return to the first column of that card and read again. The number of items correctly named in 45 s in each card was recorded and used to calculate the predicted CW score (PCW) according to the following formula: [Pcw = (W × C) / (W + C)]. Then, the PCW score was subtracted from the actual score of the CW card (number of items correctly named in the CW card) leading to obtaining the interference score (IG) as follows: IG = CW – PCW [61]. The higher IG scores indicated a better ability to inhibit interference and better cognitive function [61].

#### Choice reaction time (CRT)

Simple and choice reaction time is an important measure of visuomotor and cognitive performance. It is an important cognitive component related to sports performance, being able to discriminate among sport types and competitive levels of athletes [62, 63]. We used the Visual Choice Reaction Time Apparatus (Model 63,035 A, Lafayette Instrument Company, Indiana, USA), similar to a previous study using tDCS and endurance exercise performance in hypoxia [30]. A four-choice compatible stimulus-response paradigm was used. Participants sat comfortably in a chair in front of the response panel having four lights and corresponding response buttons beneath each light. Five visual stimuli (lights turning on) were manually given to the participants, and they were instructed to respond as quickly as they could by pushing the corresponding button on the response panel. The reaction time (RT) in each stimulus was recorded and the mean value of five efforts was calculated as each subject’s final score of CRT.

### Statistical analyses

Data are presented as the mean ± standard deviation (M ± SD). The normal distribution of each data set was evaluated by the Shapiro-Wilk normality test. Two-way repeated measures ANOVA (3 × 3 factorial design; 3 stimulation conditions and 3-time points) was used to analyze PP, MP, FI, EMG, and RPE at each time point. Two-way repeated measures ANOVA (3 × 2 factorial design; 3 stimulation conditions and 2-time points) was also used to analyze CWST and CRT at pre and post-time. Bonferroni post hoc test was used for the pairwise comparisons. In case of a violation in the assumption of sphericity, the Greenhouse-Geisser epsilon correction was applied. When the assumption of normality was not met (affective valence and arousal), the Friedman test was adopted and if significant results were obtained, Bonferroni correction was used for pairwise comparisons. Partial eta squared (η^*2*^_*p*_) was used as a measure of the effect size for the ANOVAs and interpreted as small (0.01–0.059), medium (0.06 to 0.139), or large (≥ 0.14). Cohen’s d calculation of the effect size was also used for pairwise comparison and interpreted as small (0.20–0.49), medium (0.50–0.79), or large (≥ 0.80). In addition, the Friedman test was used to compare tDCS-induced sensations, followed by Wilcoxon signed-rank tests were conducted with a Bonferroni correction for pair-wise comparisons (0.05/3 = Bonferroni corrected p = 0.017), in case of significant differences. The statistical analyses were performed using SPSS 23 (SPSS Inc., Chicago, IL, USA) and *p*˂0.05 was adopted.

## Results

### tDCS-induced sensations and blinding

All 15 participants received the experimental conditions according to the randomization. There were no serious side or adverse effects reported. The most common sensations reported were itching and burning. Pain and warmth/heat was reported but at a low frequency (< 15% of participants). No other sensation beyond the ones included in the questionnaire was reported. The location of the sensations was on the head for all participants, starting at the beginning of the stimulation, and stopping either at the beginning or middle of the stimulation *(*Table 2*)*. A significant difference among conditions was found for the discomfort, but post hoc analysis found no difference in pairwise comparisons. All participants reported these sensations to positively affect their performance. The percentage of correct guesses regarding the tDCS condition differed among conditions (χ2[2] = 30.0; p < 0.001), with DLPFC (100%; p < 0.001) and M1 (100%; p < 0.001) conditions different from sham (0%). This was because all individuals (100%) thought they were stimulated in all three conditions (active stimulation guess rates), without difference among them (χ2[2] = not applicable; p = not applicable). Hence, considering the similar tDCS-induced sensations and active guess rate it can be assumed that the study blinding protocol was effective. The overall results of the study variables are presented in Table 3.

**Table 2:** tDCS-induced sensations and the general sensation index (discomfort) felt by participants (n = 15)

Sensation		DLPFC a-tDCS				M1 a-tDCS				Sham tDCS				*χ2*	*p*
		Mean ± SD	Median (IQR)	n(%)		Mean ± SD	Median (IQR)	n(%)		Mean ± SD	Median (IQR)	n(%)			
**Itchiness**		1.20 ± 0.41	1.0 (1.0–1.0)	15 (100)		1.20 ± 0.41	1.0 (1.0–1.0)	15 (100)		1.20 ± 0.41	1.0 (1.0–1.0)	15 (100)		0.00	1.00
**Pain**		0.00 ± 0.00	0.0 (0.0–0.0)	0 (0)		0.07 ± 0.26	0.0 (0.0–0.0)	1 (6.7)		0.00 ± 0.00	0.0 (0.0–0.0)	0 (0)		2.00	0.37
**Burning**		0.40 ± 0.51	0.0 (0.0–1.0)	6 (40.0)		0.47 ± 0.52	0.0 (0.0–1.0)	7 (46.7)		0.13 ± 0.35	0.0 (0.0–0.0)	2 (13.3)		4.20	0.12
**Warmth/Heat**		0.13 ± 0.35	0.0 (0.0–0.0)	2 (13.3)		0.07 ± 0.26	0.0 (0.0–0.0)	1 (6.7)		0.00 ± 0.00	0.0 (0.0–0.0)	0 (0)		2.00	0.37
**Pinching**		0.00 ± 0.00	0.0 (0.0–0.0)	0 (0)		0.00 ± 0.00	0.0 (0.0–0.0)	0 (0)		0.00 ± 0.00	0.0 (0.0–0.0)	0 (0)		N/A	N/A
**Iron taste**		0.00 ± 0.00	0.0 (0.0–0.0)	0 (0)		0.00 ± 0.00	0.0 (0.0–0.0)	0 (0)		0.00 ± 0.00	0.0 (0.0–0.0)	0 (0)		N/A	N/A
**Fatigue**		0.00 ± 0.00	0.0 (0.0–0.0)	0 (0)		0.00 ± 0.00	0.0 (0.0–0.0)	0 (0)		0.00 ± 0.00	0.0 (0.0–0.0)	0 (0)		N/A	N/A
**Other**		0.00 ± 0.00	0.0 (0.0–0.0)	0 (0)		0.00 ± 0.00	0.0 (0.0–0.0)	0 (0)		0.00 ± 0.00	0.0 (0.0–0.0)	0 (0)		N/A	N/A
**Discomfort**		1.73 ± 0.59	2.0 (1.0–2.0)	-		1.80 ± 0.86	2.0 (1.0–2.0)	-		1.33 ± 0.49	1.0 (1.0–2.0)	-		7.20	0.03
**Start**		1.00 ± 0.00	1.0 (1.0–1.0)	-		1.00 ± 0.00	1.0 (1.0–1.0)	-		1.00 ± 0.00	1.0 (1.0–1.0)	-		N/A	N/A
**End**		1.20 ± 0.41	1.0 (1.0–1.0)	-		1.40 ± 0.51	1.0 (1.0–2.0)	-		1.27 ± 0.46	2.0 (1.0–2.0)	-		2.00	0.37
**Affect performance**		2.67 ± 0.48	3.0 (2.0–3.0)	15 (100)		2.67 ± 0.49	3.0 (2.0–3.0)	15 (100)		2.67 ± 0.49	3.0 (2.0–3.0)	15 (100)		0.00	1.00

**Note**. tDCS = transcranial direct current stimulation; DLPFC = dorsolateral prefrontal cortex; M1 = primary motor cortex; mean ± standard deviation; median (interquartile range); n(%) = indicates the number and percentage of participants who experienced a particular sensation

**Table 3:** Mean values of the study variables at specified time points under 3 different stimulation conditions (n_=_ 15)

Variables	Experimental Conditions											
	M1 Mean (SD)				DLPFC Mean (SD)				Sham Mean (SD)			
	B1	B2		B3	B1	B2		B3	B1	B2		B3
**Peak Power**_**(Watt)**_	788.8 (175.9)	747.3 (123.2)		631.5 (97.1)	795.6 (180.8)	746.3 (152.4)		707.5 (152.4)	791.3 (182.9)	754.5 (190.6)		637.9 (158.8)
**Mean Power**_**(Watt)**_	546.3 (102.5)	506.9 (61.7)		445.4 (47.1)	565.3 (98.2)	506.1 (84)		477.1 (78.2)	568.7 (102)	507.2 (71.9)		437.4 (66.4)
**Fatigue Index**_**(%)**_	59.74 (10.4)	60.6 (8.5)		63.0 (10.5)	56.7 (11.0)	60.7 (11.6)		61.6 (9.6)	57.9 (10.8)	62.5 (10.8)		69.0 (8.1)
**Heart Rarte**_**(Beats Per Min)**_	162.4 (7.1)	167.8 (6.3)		177.8 (4.2)	159.5 (9.0)	166.2 (9.5)		173.8 (9.0)	163.0 (9.0)	170.5 (8.9)		179.8 (4.9)
**RPE**_**(0−100, Borg Scale)**_	63.6 (15.0)	81.33 (14.5)		97.3 (13.6)	53.6 (16.7)	73.7 (19.9)		87.0 (19.5)	61.3 (15.1)	80.6 (18.5)		99.3 (10.4)
**EMG of VL**_**(% of MIVC)**_	49.0 (19.2)	46.4 (19.9)		45.2 (20.7)	57.9 (14.2)	57.8 (16.9)		54.3 (16.9)	44.9 (13.4)	41.1 (10.7)		38.2 (11.3)
**EMG of VM**_**(% of MIVC)**_	59.44 (24.3)	57.2 (23.6)		52.7 (21.6)	69.8 (23.8)	66.2 (21.7)		61.5 (15.6)	58.4 (23.5)	56.5 (24.4)		49.7 (19.1)
**EMG of RF**_**(% of MIVC)**_	33.4 (17.6)	29.1 (14.0)		29.0 (14.4)	40.3 (14.4)	36.1 (13.7)		32.8 (12.6)	33.1 (13.4)	30.3 (12.7)		28.4 (13.1)
**Affective Valence**_**(−5 to +5, Feeling Scale)**_	2.86 (2.0)	1.66 (1.9)		0.73 (1.6)	2.73 (1.6)	1.6 (2.4)		0.93 (2.4)	2.53 (1.5)	1.0 (1.3)		− 0.6 (1.6)
**Arousal**_**(1 to 6, Felt Arousal Scale)**_	4.53 (1.1)	3.66 (1.0)		3.0 (0.9)	4.6 (1.2)	3.66 (1.1)		2.93 (1.2)	4.4 (1.5)	3.6 (1.3)		2.4 (0.9)
	**Baseline**		**Post**		**Baseline**		**Post**		**Baseline**		**Post**	
**CWST**_**(IG score)**_	3.76 (10.2)		8.58 (8.7)		4.73 (9.5)		12.49 (10.7)		4.92 (11.0)		5.02 (11.1)	
**CRT**_**(Milliseconds)**_	414.6 (55.1)		399.3 (42.8)		407.3 (59.4)		404.0 (55.5)		410.0 (49.2)		426.8 (51.2)	

**Note**. EMG = electromyography; VL = vastus lateralis muscle; VM = vastus medialis muscle; RF = rectus femoris muscle; MIVC = maximum isometric voluntary contraction; CRT = choice reaction time; CWST = color-word Stroop test; RPE = ratings of perceived exertion; B1 = first bout; B2 = second bout; B3 = third bout

### Anaerobic performance

**Fig. 3:**
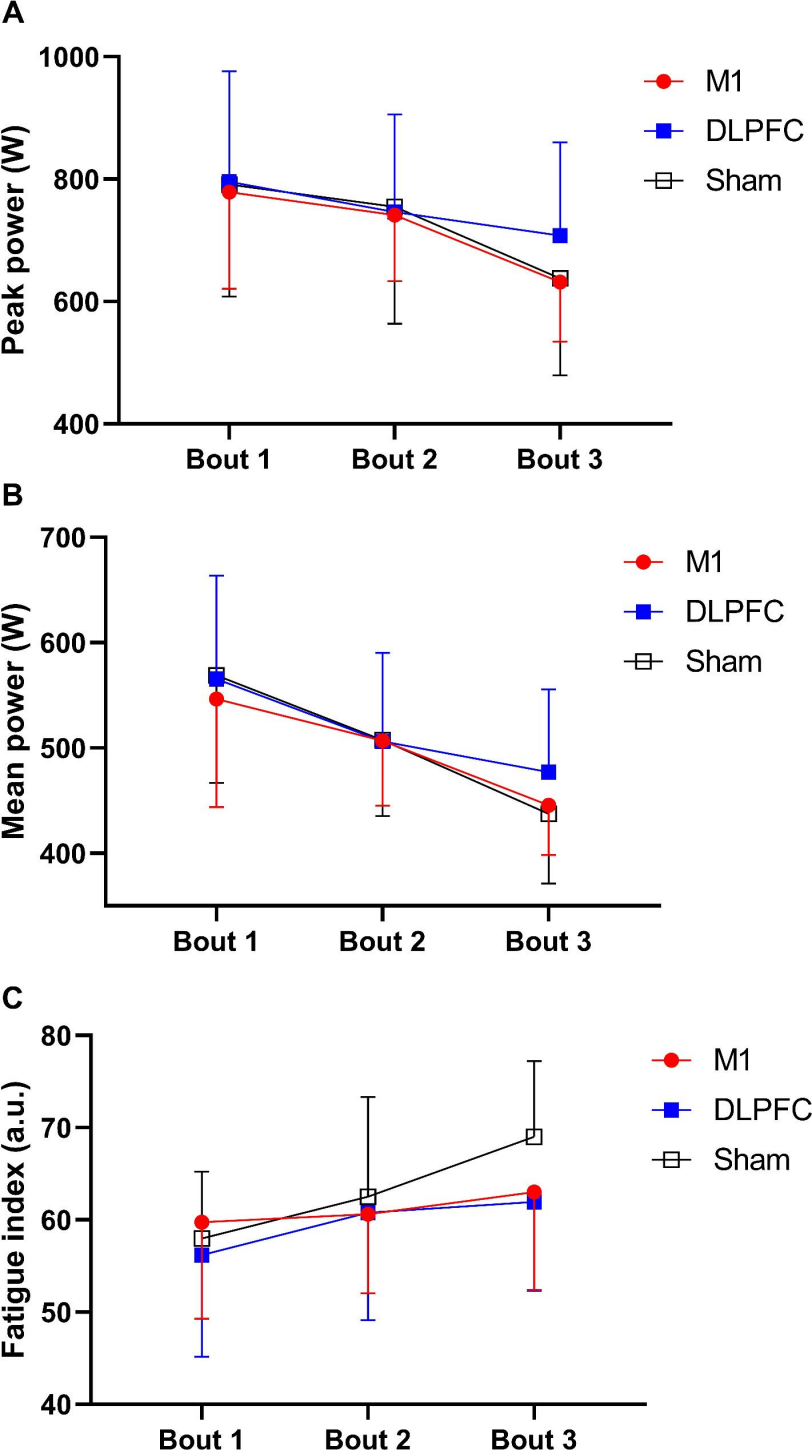
Mean values of the PP, MP, and FI during each bout of the Wingate test. ***(A)*** Peak Power (PP); ***(B)*** Mean Power (MP); ***(C)*** Fatigue Index (FI), with transcranial direct current stimulation targeting the motor cortex (M1), dorsolateral prefrontal cortex (DLPFC), and sham conditions

There was a significant main effect of time on the **PP***(F*_*(1.1,16.3)=*_*26.5, p*_*=*_*0.0001, *η^*2*^_*p =*_*0.655, Power*_*=*_*0.999)*, indicating a decrement in the **PP** over time *(See* Fig. 3A*)*, with no significant main effect of condition *(F*_*(2,28)=*_*1.11, p*_*=*_*0.34, *η^*2*^_*p =*_*0.074, Power*_*=*_*0.225)* or ‘condition × time’ interaction *(F*_*(4,56)=*_*1.65, p*_*=*_*0.17, *η^*2*^_*p=*_*0.106, Power*_*=*_*0.478)*. Likewise, there was a significant main effect of time *(F*_*(1.1,15.8)=*_*51.7, p*_*=*_*0.0001, *η^*2*^_*p =*_*0.787, Power*_*=*_*1.0)*, demonstrating that the **MP** decreased over time *(See* Fig. 3B*)*, with no significant main effect of condition *(F*_*(2,28)=*_*1.13, p*_*=*_*0.33, *η^*2*^_*p =*_*0.075, Power*_*=*_*0.229)* or ‘condition × time’ interaction *(F*_*(1.9,27.1)=*_*1.9, p*_*=*_*0.1, *η^*2*^_*p =*_*0.125, Power*_*=*_*0.371)*. Finally, there was a significant main effect of time on **FI***(F*_*(1.4,20.2)=*_*15.1, p*_*=*_*0.0001, *η^*2*^_*p =*_*0.520, Power*_*=*_*0.988)*, showing that the **FI** increased over time *(See* Fig. 3C*)*, with no significant main effect of condition *(F*_*(2,28)=*_*1.27, p*_*=*_*0.29, *η^*2*^_*p =*_*0.084, Power*_*=*_*0.254)* or ‘condition × time’ interaction effect *(F*_*(2.3,32.2)=*_*1.57, p*_*=*_*0.19, *η^*2*^_*p =*_*0.101, Power*_*=*_*0.331)*.

### Physiological responses

**Fig. 4:**
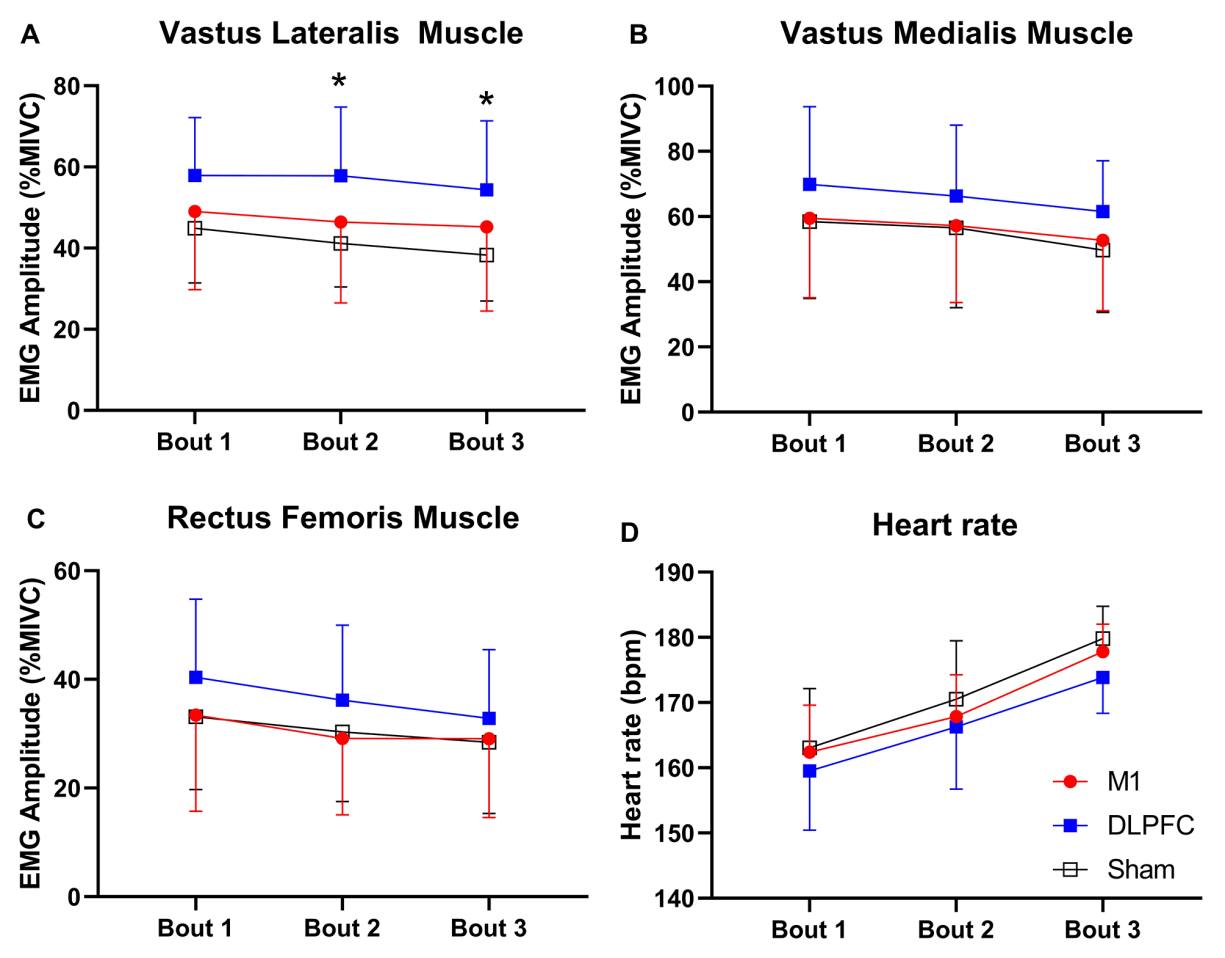
Mean values of the EMG amplitude and HR during each bout of the Wingate test. ***(A)*** EMG amplitude of the Vastus Lateralis (VL) muscle; ***(B)*** EMG amplitude of the Vastus Medialis (VM) muscle; ***(C)*** EMG amplitude of the Rectus Femoris (RF) muscle; (D) heart rate, with transcranial direct current stimulation targeting the motor cortex (M1), dorsolateral prefrontal cortex (DLPFC), and sham conditions. * = Significant difference between DLPFC and sham conditions

There was a significant main effect of time on the **EMG** of the VL muscle *(F*_*(2,28)=*_*10.4, p*_*=*_*0.0001, *η^*2*^_*p =*_*0.428, Power*_*=*_*0.979)*, and a main effect of condition *(F*_*(2,28)=*_*4.1, p*_*=*_*0.027, *η^*2*^_*p =*_*0.227, Power*_*=*_*0.680)*, with no significant ‘condition × time’ interaction *(F*_*(2.5,35.4)=*_*1.2, p*_*=*_*0.32, *η^*2*^_*p =*_*0.079, Power*_*=*_*0.273)*. Although the **EMG** of the VL decreased over time in all three conditions *(See* Fig. 4A*)*, pairwise comparisons revealed that the **EMG** of the VL muscle was significantly higher under the DLPFC condition compared to the sham condition at both the second *(p*_*=*_*0.016, d*_*=*_*1.2, Δ*_*=*_*40.3%)* and third bouts (*p*_*=*_*0.016, d*_*=*_*1.1, Δ*_*=*_*42.1%)* of the Wingate test. There was a significant main effect of time on the **EMG** of the VM *(F*_*(2,28)=*_*15.5, p*_*=*_*0.0001, *η^*2*^_*p =*_*0.526, Power*_*=*_*0.998)* with no significant main effect of condition *(F*_*(2,28)=*_*1.16, p*_*=*_*0.32, *η^*2*^_*p =*_*0.077, Power*_*=*_*0.278)* or ‘condition × time’ interaction *(F*_*(2.3,32.5)=*_*0.26, p*_*=*_*0.89, *η^*2*^_*p =*_*0.019, Power*_*=*_*0.091;* Fig. 4B*)*. Similarly, There was a significant main effect of time on the **EMG** of the RF *(F*_*(2,28)=*_*14.5, p*_*=*_*0.0001, *η^*2*^_*p =*_*0.509, Power*_*=*_*0.997)* with no significant main effect of condition *(F*_*(4,56)=*_*0.71, p*_*=*_*0.58, *η^*2*^_*p =*_*0.049, Power*_*=*_*0.217)* or ‘condition × time’ interaction *(F*_*(2,28)=*_*1.41, p*_*=*_*0.25, *η^*2*^_*p =*_*0.092, Power*_*=*_*0.234;* Fig. 4C*)*.

There was a significant main effect of time on the **HR***(F*_*(2,28)=*_*113.01, p*_*=*_*0.0001, *η^*2*^_*p =*_*0.890, Power*_*=*_*0.988)*, indicating that the **HR** increased over time *(See* Fig. 4D*)*, with no significant main effect of condition *(F*_*(2,28)=*_*3.25, p*_*=*_*0.054, *η^*2*^_*p =*_*0.188, Power*_*=*_*0.254)* or ‘condition × time’ interaction effect *(F*_*(4,56)=*_*1.02, p*_*=*_*0.4, *η^*2*^_*p =*_*0.068, Power*_*=*_*0.331).*

### Psychophysiological responses

**Fig. 5:**
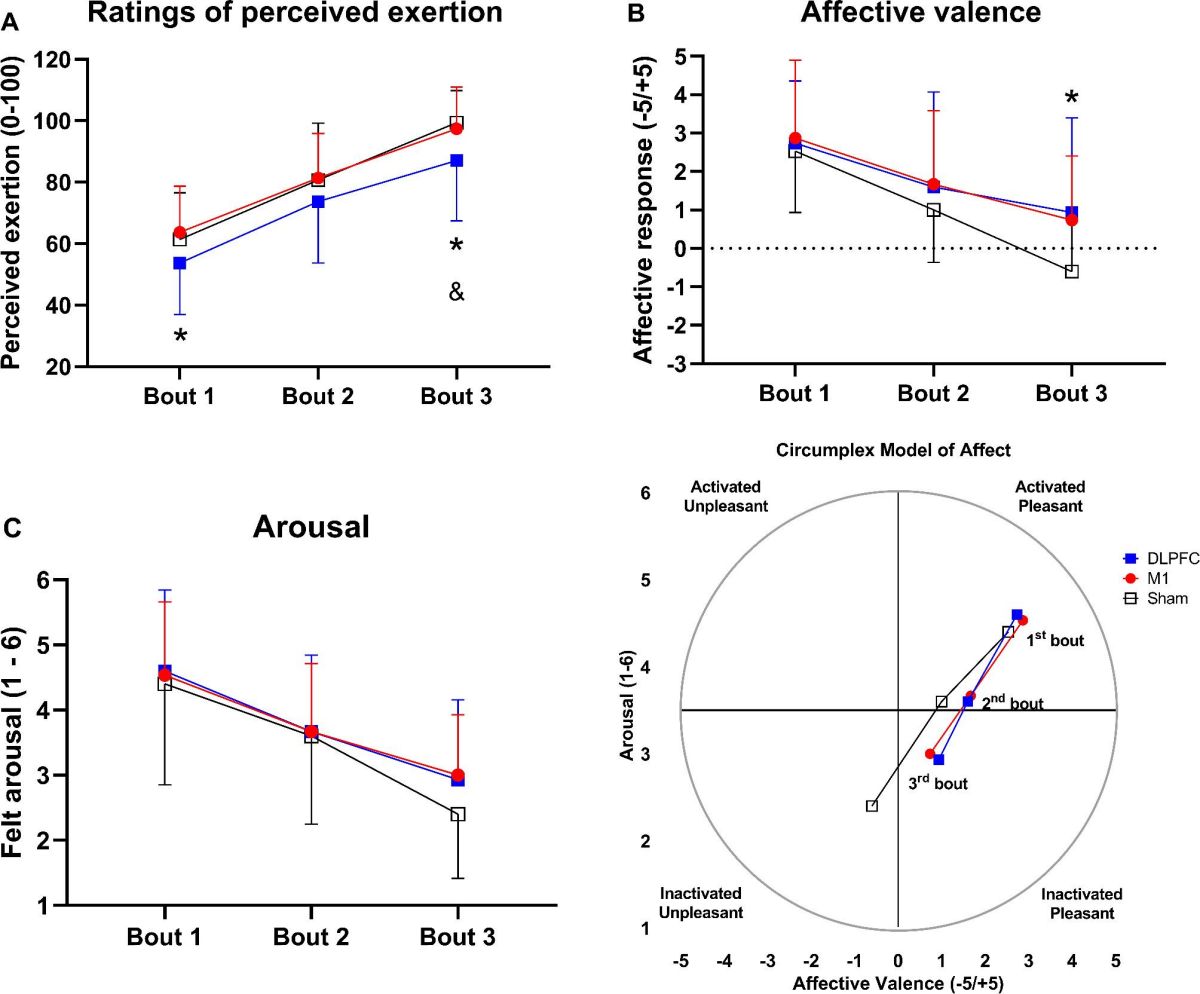
Psychophysiological responses during each bout of the Wingate test. RPE (Borg 0-100 scale), affective valence (FS scale), and arousal (FAS scale) were recorded at min 2 of each 4 min active recovery performed after each bout of the 30-s Wingate test under 3 experimental conditions. FS: Feeling scale; FAS: Felt arousal scale; RPE: ratings of perceived exertion. * = Significant difference between DLPFC and sham conditions; & = Significant difference between DLPFC and M1 conditions

There was a significant main effect of condition on the **RPE***(F*_*(2,28)=*_*4.17, p*_*=*_*0.026, *η^*2*^_*p =*_*0.230, Power*_*=*_*0.687)*, and a main effect of time was (*F*_*(1.4,19.7)=*_*56.2, p*_*=*_*0.0001, *η^*2*^_*p =*_*0.801, Power*_*=*_*1.0)*, but no significant ‘condition × time’ interaction *(F*_*(4,56)=*_*0.52, p*_*=*_*0.7, *η^*2*^_*p =*_*0.036, Power*_*=*_*0.167)*. While **RPE** increased over time in all conditions *(See* Fig. 5A*)*, pairwise comparisons revealed that the **RPE** was significantly lower under the DLPFC condition in the first bout (*p*_*=*_*0.048, d*_*=*_*0.48, Δ*_*=*_*-12.5%)* compared to the sham *and* in third bout of the Wingate test compared to both M1 (*p*_*=*_*0.003, d*_*=*_*0.62, Δ*_*=*_*-10.5%)* and sham (*p*_*=*_*0.047, d*_*=*_*0.82, Δ*_*=*_*-12.38%)* conditions.

There was no difference in the **affective valence** in the first *(χ*^*2*^_*(2)=*_*1.05, p*_*=*_*0.52)* and second bouts *(χ*^*2*^_*(2)=*_*3.79, p*_*=*_*0.15)*, but a significant difference was found among the conditions in the third bout of the Wingate test *(χ*^*2*^_*(2)=*_*9.38, p*_*=*_*0.009).* Pairwise comparisons showed that the **affective valence** was significantly higher in the DLPFC condition compared to the sham condition after the third bout of the Wingate test (*p*_*=*_*0.032, d*_*=*_*0.77).* Moreover, an effect of time was observed for the M1 *(χ*^*2*^_*(2)=*_*23.13, p*_*=*_*0.0001)*, DLPFC *(χ*^*2*^_*(2)=*_*14.29, p*_*=*_*0.001)*, and Sham *(χ*^*2*^_*(2)=*_*18.77, p*_*=*_*0.0001)* conditions, indicating a decrease in **affective valence** over time *(See* Fig. 5B*)*.

Similarly, while there was no difference in **arousal** in the first *(χ*^*2*^_*(2)=*_*1.43, p*_*=*_*0.48)* and second bouts *(χ*^*2*^_*(2)=*_*0.86, p*_*=*_*0.64)*, a significant difference was found among the conditions after the third bout of the Wingate test *(χ*^*2*^_*(2)=*_*7.77, p*_*=*_*0.021).* However, no specific difference among the conditions was observed after Bonferroni’s adjustment *(p> 0.05)*. An effect of time in **arousal** was also found for the M1 *(χ*^*2*^_*(2)=*_*20.53, p*_*=*_*0.0001)*, DLPFC *(χ*^*2*^_*(2)=*_*19.88, p*_*=*_*0.0001)*, and Sham *(χ*^*2*^_*(2)=*_*18.68, p*_*=*_*0.0001)* conditions showing that the **arousal** decreased over time *(See* Fig. 5C*)*.

Finally, the analysis of the Circumplex Model of Affect showed that in the first and second bout of the Wingate test participants stayed in the upper right quadrant (activated-pleasant), but in the third bout participants moved to the lower right quadrant (inactivated-pleasant) in the M1 and DLPFC tDCS conditions, while they moved to the upper left quadrant (inactivated-unpleasant) in the sham condition *(*Fig. 5D*)*.

### Cognitive function

**Fig. 6:**
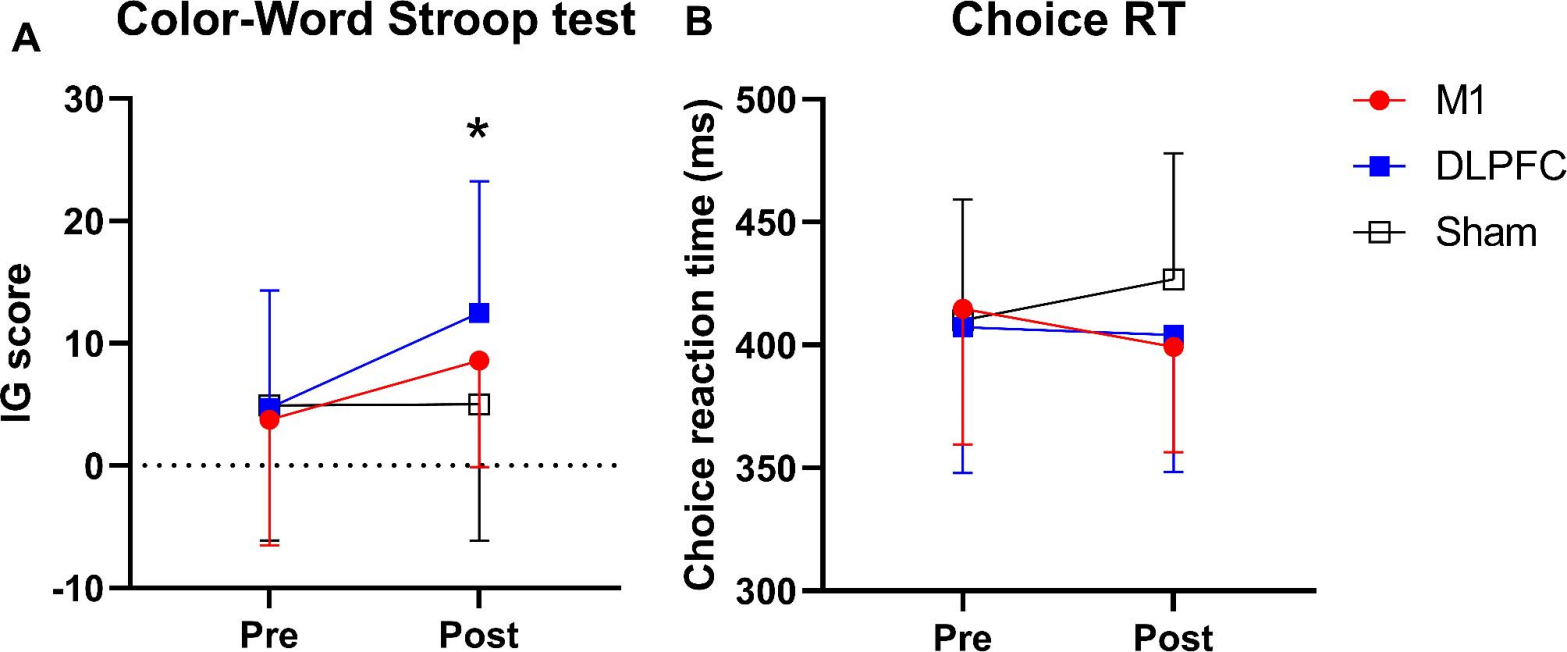
Cognitive performance at baseline and after the whole Wingate test procedure. ***(A)*** Color-Word Stroop Test (CWST); ***(B)*** Choice Reaction Time (CRT), with transcranial direct current stimulation targeting the motor cortex (M1), dorsolateral prefrontal cortex (DLPFC), and sham conditions. * = Significant difference between DLPFC and sham conditions

There was a significant main effect of time on the **CWST** values *(F*_*(1,14)=*_*9.5, p*_*=*_*0.008, *η^*2*^_*p =*_*0.405, Power*_*=*_*0.818)* and ‘condition × time’ interaction *(F*_*(2,28)=*_*5.62, p*_*=*_*0.009, *η^*2*^_*p =*_*0.287, Power*_*=*_*0.819)* with no significant main effect of condition *(F*_*(2,28)=*_*1.2, p*_*=*_*0.31, *η^*2*^_*p =*_*0.08, Power*_*=*_*0.242)*. While CWST performance improved from pre to post in all conditions, pairwise comparisons indicated that the **CWST** was significantly higher under the DLPFC condition compared to the sham condition (*p*_*=*_*0.04, d*_*=*_*0.68, Δ*_*=*_*147%;* Fig. 6A*)* after performing the exercise protocol (post-test). Finally, there was no significant main effect of time *(F*_*(1,14)=*_*0.017, p*_*=*_*0.89, *η^*2*^_*p =*_*0.001, Power*_*=*_*0.052)*, condition *(F*_*(2,28)=*_*2.01, p*_*=*_*0.15, *η^*2*^_*p =*_*0.126, Power*_*=*_*0.380)* or ‘condition × time’ interaction *(F*_*(1.3,19.1)=*_*2.95, p*_*=*_*0.06, *η^*2*^_*p =*_*0.176, Power*_*=*_*0.432)*, on the **CRT***(See* Fig. 6B*)*.

## Discussion

Here we compared, for the first time, the effect of anodal-tDCS over M1 and DLPFC areas on repeated all-out anaerobic performance, psychophysiological responses, and cognitive function. The main findings of this study were that tDCS did not affect repeated anaerobic performance, HR, felt arousal, CRT, and EMG activity of VM and RF muscles. However, tDCS targeting the left DLPFC significantly decreased RPE, increased EMG activity of VL muscle, and improved the affective valence and CWST score.

So far, few studies have explored the efficacy of tDCS for anaerobic performance, in particular, anaerobic performance with repeated nature (repetitions ranging from <10 to 90 s in duration) which is quite similar to the performance in many sports [25, 27]. In this context, Sasada et al. [23] showed that anodal tDCS over M1 (2 mA for 15-min) did not improve sprint performance on an 8-s or 30-s maximal-effort cycling task in athletes compared to sham. They concluded that while tDCS can modulate the neural population involved in regulating sub-maximal endurance performance, it seems that the neural population involved in generating sprint performance is not affected by tDCS over the M1 area [23].

In the present study, anaerobic performance gradually decreased over time but, contrary to our main hypothesis, anodal tDCS over the M1 or left DLPFC had no significant effect on repeated all-out performance (i.e., PP, MP, and FI). It has been postulated that voluntaryly recruiting all motoneuron pools at their highest firing frequency is crucial for reaching peak performance in short all-out activities [64]. During the repeated all-out tasks, however, as more bouts are performed, an individual’s ability to maintain peak performance decline due to the development of both central and peripheral fatigue [28]. It has been suggested that while peripheral fatigue accounts for the early reduction in performance in high-intensity exercise, central fatigue contributes the most toward the end of the exercise [65]. This concept might help to explain why tDCS had no impact on anaerobic performance in the current investigation. It appears that peripheral fatigue was the primary cause of performance decline, and as a result, tDCS was unable to have a significant impact, presumably because the CNS was still operating at its best capacity to activate the target muscles.

Recent findings have highlighted the role of the PFC and particularly, the DLPFC area in regulating different aspects of exercise performance [3, 5, 8, 66]. In this context, Robertson and Marino [9] proposed that the PFC (in particular its lateral region) would be involved in exercise tolerance and termination, along with other brain areas such as the anterior cingulate cortex, premotor area, and orbitofrontal cortex creating the pathways for interpreting afferent signals coming from different parts of the periphery. In this case, the PFC has been proposed to play a substantial role in integrating sensory afferent signals and providing suitable responses in a hierarchical manner leading to overruling inhibitory inputs and maintaining motor output [5, 12]. In this regard, Angius et al. [18] reported that anodal tDCS targeting the left DLPFC improved endurance cycling performance with decreased RPE. It appears that in the current study, during the second and third bouts of the Wingate test, when afferent inhibitory signals from the working muscles and the CNS itself arrived more intensively in the PFC, stimulating the left DLPFC was able to increase the activity of this region, resulting in an improvement in the information processing of those afferent signals and a lower RPE during the active recovery after the Wingate test. Nevertheless, this lowered RPE was not accompanied by a significantly lower HR in the DLPFC condition compared to the sham condition. Indeed, there is no consensus on the effectiveness of different montages of tDCS on cardiovascular responses at rest and during exercise [67]. Some studies have shown that anodal tDCS over the temporal cortex reduced HR during exercise probably via increasing the parasympathetic activity while other studies reported no significant effects of tDCS on HR [67–69]. Our results are in line with most of the studies showing no significant effect of tDCS on HR. It seems that in the present study, the target areas in the brain (M1 and DLPF), which are not considered to be profoundly involved in the regulation of the cardiovascular system during exercise, and also the tDCS montage are the main reasons for no significant positive effect of tDCS on HR.

Our results demonstrated that after performing the third bout of the Wingate test, the affective valence was higher in the DLPFC tDCS compared to the sham which shows a possible moderating effect of the DLPFC stimulation for perceived pleasure [35]. Interestingly, when affective valence and arousal were used in the circumplex model of affect, which has recently been considered a viable tool for evaluating the perceptual responses [54], we observed that the perceptual responses were similar in the M1, DLPFC, and sham conditions after the first and second bout of the Wingate test being in the “Activated-Pleasant” quadrant. After the third bout, however, the perceptual responses under the M1 and DLPFC conditions moved to the “Unactivated-Pleasant” quadrant while in the sham condition, the perceptual responses shifted towards the “Unactivated-Unpleasant” quadrant. More support for our results can be provided by Rodrigues et al. [34] showing that anodal tDCS over the DLPFC could maintain the perceived pleasure despite an increase in the EMG activity of the target muscle. This provides additional support for the surmise that the tDCS could probably impose a modulatory effect on short all-out performance by regulating the perceptual responses assessed qualitatively using the circumplex model of affect.

The results also showed that there was no difference in the EMG amplitude of the VM and RF muscles under 3 different stimulation conditions but, conversely, the EMG amplitude of the VL muscle was higher during the second and third bouts of the Wingate test under the DLPFC condition compared to the sham condition. Previous studies have reported conflicting results concerning the causative effect of tDCS on muscle EMG as most of the studies did not find any effect of tDCS on the muscle EMG [18, 70, 71] while some recent findings suggest that tDCS might affect EMG [19, 30, 66, 72, 73]. In this sense, it has been suggested that the tDCS-induced change in the excitability of target regions, which in turn might alter the motor unit recruitment strategies in the brain, is probably the main mechanism by which tDCS could induce its effect on muscle activity reflected by EMG [19, 72]. Surprisingly, while in previous studies anodal tDCS of M1 has yielded positive effects on muscle EMG, in the present study, we saw a higher EMG amplitude in VL muscle in the DLPFC condition compared to sham, but not in the M1 tDCS. It’s not clear why the EMG amplitude was not affected by the M1 tDCS in the present study. One reason might be the fact that we did not use transcranial magnetic stimulation (TMS) which is the gold standard method for hot spotting the precise region representing the motor area of the lower limb over the M1 for tDCS [74]. It is worth mentioning, however, that the international 10–20 EEG system has been corroborated as a valid method for stimulating target areas in the brain in previous studies [12, 21, 47, 66, 73]. Furthermore, previous studies have highlighted the substantial role of the group III and IV muscle afferent in regulating the central motor drive to the working muscle by providing inhibitory feedback to the different regions of the CNS during exercise [2, 6]. Those studies have also indicated that even though the precise sites in the CNS receiving nociceptive muscle afferents are still unknown, upstream neural circuits from M1, as well as the motor cortex itself, are the probable sites [6, 75–77]. It can be speculated that the PFC may override the inhibitory inputs and maintain motor output [3, 5, 8, 9, 12], which suggests that during repeated short all-out activities, in particular toward the end of the task, when inhibitory afferent feedback arriving at the CNS is stronger and motor cortex is working at its highest level to fulfill the demands of such intense tasks, stimulating the DLPFC area could modulate the inhibitory sensory information and, likely, increase the net motor output to the periphery as reflected in the higher EMG activity. Nonetheless, more research is warranted to corroborate this claim.

It has also been shown that the effect of acute physical activity on cognitive function depends primarily on exercise modalities and the cognitive task performed and accordingly, inconsistent results have been reported in the previous studies [78]. In the present study, it seems that performing 3 bouts of the Wingate test had no detrimental effects on cognitive function as measured by CWST and CRT which is in line with some of the previous studies [79]. It must be noted that in the present study, cognitive tests were performed after 2 min of active recovery following the third bout of the Wingate test which might have reduced the cognitive burden of the repeated all-out task. Moreover, since the cognitive tests were first performed at the baseline in each session, the learning effect could be a possibility compensating for the detrimental effect of repeated strenuous exercise on cognitive function [80, 81]. On the other hand, we observed that after performing the whole Wingate test, there was no difference in CRT among the experimental condition while the CWST score was higher under the DLPFC condition compared to the sham condition. Strong evidence coming from structural and functional magnetic resonance imaging studies has shown that the DLPFC area is involved in inhibitory control, attention, and cognitive control [82–84]. In this context, Loftus et al. [83] reported that anodal tDCS over the left DLPFC improved the inhibitory control and speeded up the information processing during an incongruent Stroop test. Similarly, Abedanzadeh et al. [60] showed that anodal stimulation of the left DLPFC reduced the reaction time to the Stroop test and also improved the inhibitory control which is crucial for better performance in the incongruent Stroop situation. Our results are in line with these studies and, as a new finding, emphasize that anodal tDCS of the left DLPFC could improve information processing and inhibitory control after a very demanding repeated short all-out activity since no physical tasks were performed in previous studies.

Despite taking all necessary details into account to provide optimum control over the study procedure, caution must be taken when considering the findings of the present study because they are not free from the effects of limiting factors. In this study, we were not able to use TMS for hot spotting the lower limb representation in the M1 which might have affected the response of this region to tDCS. In addition, we were not able to use neurophysiological/neuroimaging measures that could provide more information concerning any changes induced in brain activity. The inclusion of a complete control condition (without any intervention, even sham) would help to rule out a placebo effect, as expectations and placebo effects might be induced in tDCS studies [40, 41]. Finally, the fact that only men were included in the present study limits the study’s generalizability to women, as sex might influence tDCS outcomes [85, 86].

## Conclusion

The results of the present study showed that anodal tDCS targeting neither M1 nor left DLPFC improved anaerobic performance during repeated all-out Wingate tests. Nevertheless, our results demonstrated the positive effects of anodal tDCS targeting the left DLPFC on RPE, EMG activity of the VL muscle, affective valence, perceptual responses (qualitatively measured through the circumplex model of affect), and also cognitive function which could have practical indications for future studies in this particular field. Future studies aiming to use brain stimulation techniques to improve anaerobic performance might test the efficacy of anodal tDCS concurrently targeting M1 and DLPFC, which has been shown to considerably increase the corticospinal excitability, transcranial pulse or altering current stimulation, and tDCS with different intensity, duration, and electrode location and size.

## Data Availability

The data generated and/or analyzed during the current study are available from the corresponding author or reasonable request.

## Abbreviations

CRT: Choice reaction time
CWST: Color-word Stroop test
DLPFC: Dorsolateral prefrontal cortex
EMG: Electromyography
FA: Felt arousal
FI: Fatigue index
HR: Heart rate
M1: Primary motor cortex
MIVC: Maximal isometric voluntary contraction
MP: Mean power
PFC: Prefrontal cortex
PP: Peak power
PS: Pleasure sensation
RF: Rectus femoris
RPE: Rating of perceived exertion
tDCS: Transcranial direct current stimulation
VL: Vastus lateralis
VM: Vastus medialis
